# Improved Functional Outcomes With No Failures Three Years After Calipered Restricted Kinematic Alignment Total Knee Arthroplasty in Japanese Patients

**DOI:** 10.7759/cureus.87654

**Published:** 2025-07-10

**Authors:** Adarsh Krishna K Bhat, Takafumi Hiranaka, Yasuhiro Fukai, Motoki Koide, Takaaki Fujishiro, Koji Okamoto

**Affiliations:** 1 Orthopedic Surgery and Joint Surgery Center, Takatsuki General Hospital, Takatsuki, JPN; 2 Orthopedic Surgery, Apollo Hospitals, Bengaluru, IND

**Keywords:** alignment, arthroplasty, calipered, kinematic, ligament balancing

## Abstract

Aims

Restricted kinematic alignment (rKA) has emerged as a safer alternative for total knee arthroplasty (TKA) in cases with extreme anatomical variations, particularly in Asian populations. However, it typically requires computer-assisted robotics and navigation. This study therefore evaluates the efficacy and safety of caliper-based rKA-TKA in a Japanese cohort without computer assistance.

Methods

We conducted a retrospective review of 67 knees in 43 patients who underwent caliper-based rKA-TKA between May 2020 and May 2021 at our hospital. Clinical outcomes were evaluated at a three-year follow-up, including Tegner activity score, Oxford knee score, Knee Society knee score and functional score, and Forgotten Joint Score 12. Radiological parameters were also assessed, such as mechanical hip-knee-ankle angle and coronal plane alignment of the knee classification.

Results

Significant improvements in clinical scores were observed at three years (p<0.001). Radiologically, 70% of knees were within the safe alignment range postoperatively (p<0.001). Notably, no revisions were required, and implant survival was 100%. Positive correlations were found between changes in mechanical hip-knee-ankle angle and improvements in Tegner activity score, Oxford knee score, and Knee Society knee score.

Conclusion

Caliper-based rKA-TKA achieved satisfactory alignment and clinical outcomes without computer assistance, demonstrating its safety and effectiveness for Japanese patients. Long-term follow-up is nonetheless warranted to confirm these findings.

## Introduction

In total knee arthroplasty (TKA), the mechanically aligned (MA) approach restores the limb alignment to neutral, positions components perpendicular to the mechanical axes, and ensures that gaps are parallel and equal in both flexion and extension. This approach emphasizes mechanical stability rather than respecting the native anatomy and kinematics. Long-term durability, accounting for 95% of 10-year survivorship in most national registries, has been accomplished [1,2]. However, patient satisfaction is not perfect: around 20% of patients are unsatisfied [3-5]. Reasons for such unfavorable results might be the alteration of alignments, joint lines, soft tissue balancing, and kinematics.

Kinematic alignment (KA) TKA has recently emerged as an alternative to MA-TKA, where the implants restore the pre-arthritic knee anatomy [6,7], aligning the components along the three kinematic axes: flexion and extension axis of the tibia (cylindrical axis), the patella (patella femoral flexion and extension), and the longitudinal tibial axis (tibia internal and external rotation), permitting kinematics more similar to those of native knees [8]. Theoretically, this improves ligament balancing and decreases the necessity for soft tissue release, as component alignment will more closely match individual knee anatomy and the corresponding soft tissue envelope of the joint [9].

However, it is still unclear whether the KA approach to TKA is suitable for all patients. Some patients have pathological alignment such as bone defects and genuine extra-articular malalignment. Restricted kinematic alignment (rKA) has been emerged to address such patients [10,11]. In rKA, the KA procedure is executed if alignments are within the safe range, otherwise components are set at a defined angle. It is considered to be safe and especially useful in Asian countries owing to the prevalence of extreme varus alignments [12,13]. The results from western countries cannot be easily applied to Japanese populations, where we think there are more varus outliers than in previous reports from Caucasian cohorts [14].

rKA typically requires computer assistance, such as in navigation, robotics, or patient-specific instrumentation [15]. Conversely, we have performed rKA by caliper technique without computer assistance by referencing bony landmarks and controlling the bone cutting thickness [16]. Here, we aim to evaluate the effectiveness and safety of CrKA-TKA (calipered restricted kinematic alignment-total knee arthroplasty) in Japanese patients. We hypothesized that CrKA controls the alignment within the safe range, with favorable clinical outcomes and without early aseptic revision.

## Materials and methods

This retrospective study was performed according to the Declaration of Helsinki and approved by the Ethics Committee of our Institution (2023-8). All procedures were carried out with informed patient consent. We studied consecutive patients who underwent primary rKA-TKA at our hospital between May 2020 and May 2021. During this period, candidates for knee arthroplasty (those with full-thickness cartilage wear or signs of osteonecrosis on at least one condyle and continuous pain and disturbance of daily living despite conservative treatment for three months or more) were screened for the eligibility for unicompartmental knee arthroplasty (UKA) (functioning anterior cruciate ligament, intact cartilage on the opposite condyle, and acceptable patellofemoral disease) using radiographic assessment tools. If a patient was deemed unsuitable for UKA, rKA-TKA was performed (around 40% of arthroplasty patients). We excluded patients who were not followed for three years, those who underwent other major surgeries such as on the hip and spine, and those who had severe diseases such as dementia.

We used either the Zimmer Persona® CR surface (Zimmer Biomet, Warsaw, IN) or Medacta GMK® Sphere CR Knee (Medacta International, Castel San Pietro, Switzerland) system. Both systems follow medial pivot designs, with multi-radius (J-shape) and single-radius designs, respectively.

We collected the patients’ preoperative and postoperative (three years) ranges of movement, patient-related outcomes measures, Tegner activity score (TAS), Oxford knee score (OKS), Japanese Orthopedic Association knee osteoarthritis treatment score (JOAS), Knee Society Score (functional score) (KSS-F), and Knee Society Score (knee score) (KSS-K).

Preoperative planning

The preoperative alignment of the limb was measured on long-standing weight-bearing scanograms, and the following angles were measured:

Mechanical hip-knee-ankle angle (mHKA): the angle between the femoral and tibial mechanical axes. A negative value indicates a varus angle, and a positive value indicates a valgus angle.

Mechanical lateral distal femoral angle (mLDFA): the angle between the femoral mechanical axis and femoral joint line.

Medial proximal tibial angle (MPTA): the angle between the tibial mechanical axis and tibial joint line. The posterior slope was also measured from the lateral radiography.

Arithmetic hip-knee-ankle angle (aHKA) was calculated as MPTA - LDFA and joint line obliquity (JLO) as MPTA + LDFA [17].

Our restriction protocol is shown in Table 1. We set the restriction boundaries as 0° to 5° varus for MPTA and LDFA and 9° for the posterior slope. As a result, aHKA becomes 0° to 5° varus. However, in extreme cases, the range was slightly enlarged to avoid significant joint change and ligament imbalance.

**Table 1:** The target boundary of restriction LDFA, lateral distal femoral angle; MPTA, medial proximal tibial angle; aHKA, arithmetic hip-knee-ankle angle

Parameters	Angles
LDFA	85° to 90°
MPTA	85° to 90°
aHKA	-5° to 3°

The boundary is widened if the correction angle is >5°.

The corrective osteotomy was performed by controlling the bone cut thickness based on the “1 mm for 1°” principle based on our previous study [16]. For example, if the LDFA is 92° (2° varus), the medial condyle was cut 5 mm (2 mm thinner than the component thickness, compensating for the cartilage wear) and the lateral condyle was cut 9 mm. If the MPTA is 83°, the medial tibial thickness is set at 6 mm (2 mm thinner than the tibial component), whereas the lateral thickness is 10 mm.

Regarding the posterior femoral condyle, the cutting is set at the same thickness as the component (9 mm for Persona, 8 mm for Sphere). However, if the posterior slope is too steep (approximately < 78°), the cutting thickness is increased by 1 mm. Likewise, if the lateral femoral condyle thickness is lessened, the lateral posterior cutting thickness is reduced by the same thickness.

Operative procedures

All patients were operated upon under general anesthesia along with a single shot femoral triangle block, with arthrotomy performed using minimally invasive muscle-sparing subvastus approach as described previously [18]. After joint exposure and osteophyte removal (both on the tibial and femoral sides), an intramedullary rod is inserted into the medullary canal. The paddle is further positioned against the distal femoral condyles, and distal femoral osteotomy can be made 9 mm from the cartilage surface. If there is a cartilage defect, the distal femoral cut is instead made 7 mm from the surface, with 2-mm correction attributed to the cartilage loss.

No further adjustments for distal femoral cuts are required in most cases. In cases with excessive femoral defect or extreme original alignment, further adjustment in the osteotomy angle is needed using a metal plate inserted between the articular surface and the paddle. The LDFA angle can be decreased or increased by keeping the metal plate on the medial or lateral condyle, respectively.

It is assumed that a 1° change in the LDFA corresponds to 1-mm bone cut based on the "1 mm for 1°" principle mentioned earlier [16]. The metal plate used is usually an angel wing or a saw blade with a 2-mm thickness.

When Persona system is used for the osteotomy, a dial guide is used in cases requiring femoral correction. First, an intramedullary rod is inserted (described above), followed by placing the osteotomy guide, with a 2-mm metal plate placed on the side with worn cartilage to compensate for the cartilage defect. Measurements from the guide are then recorded when the paddles on both sides make contact with the bone articular surface and the metal plate. The thickness of the bone that will be cut can be rechecked using an adjustable stylus, which should be 9 mm on the normal side and 7 mm on the worn side.

After the distal femoral cut, the bone fragments are measured for their thickness using Vernier calipers, and it is confirmed whether they match with the preoperative planning.

Posterior femoral condyle osteotomy is then performed. The osteotomy should be equivalent to the thickness of the femoral component with no external rotation, as our principle is strictly to resurface. Most components have posterior condyle measurement corresponding to 8 mm for Medacta GMK® or 9 mm for Zimmer Persona®. If there is wear of the bone/cartilage, then osteotomy is performed by applying adequate rotation using the metal plate.

By preference, we use anterior reference, after which the posterior condylar cuts are taken and the bone fragments measured. If there is a margin of error ≥2 mm, a recut can be planned without affecting anterior osteotomy surface. An adjustable stylus can be used to assess the thickness of the posterior condyles as well as the distal femur before performing the osteotomy.

Tibial osteotomy

**Figure 1:**
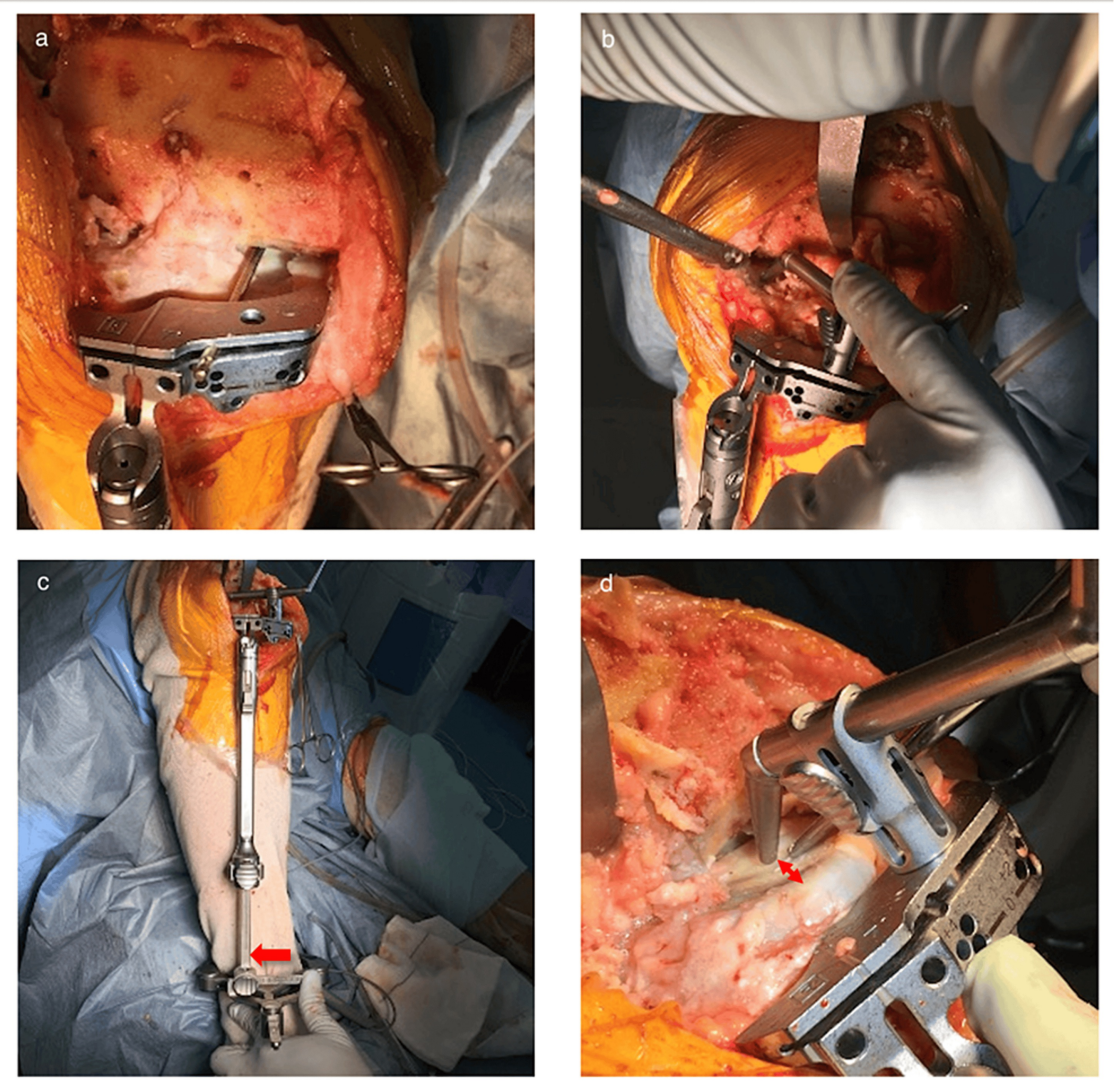
Procedure of tibial osteotomy a. A pin or angel wing penetrating the slot of the tibial cutting block is adjusted to be parallel to the medial tibial plateau. b. Maintaining the posterior slope, a 10-mm stylus is set at the center of the lateral tibial plateau. c. The ankle piece of the extramedullary rod is shifted laterally (red arrow) but never exceeds the lateral malleolus to restrict the varus angle of the tibial cutting surface up to 5.5°. d. The inclination of the extramedullary rod is adjusted so that there is a 2-mm space between the tip of the 10-mm stylus and the medial tibial bone surface (red arrow).

Fundamentally, the tibial cut is taken parallel to the tibial articular surface. Using the extramedullary tibial guide, the center of the proximal guide is aligned with the center of tibial intercondylar eminence. Next, the posterior slope is adjusted. The adjustment of the posterior tilt is done using either an angel wing or a pin placed through the slot in the persona cutting block. Posterior tilt is fine-tuned to be parallel to the joint alignment plane. If the slope is too steep, the posterior slope is restricted up to 9°. A 10-mm stylus is positioned at the lateral plateau tibia, and the alignment rod of the extramedullary guide is moved laterally to cut the proximal tibia in the varus (varus osteoarthritis knee) while preserving the cut thickness. The thickness of the medial plateau is then adjusted with an adjustable stylus to 8 mm, referring to the residual cartilage margin (Figure 1). The simultaneous use of two styluses of 10 mm and 8 mm on the lateral and the medial plateaus, respectively, makes it easier to cut the proximal tibia in the right inclination. The lateral malleolus is kept as a landmark to restrict the varus cut to an angle of 5.5° [19].

After the bony cut, the medial and lateral gaps are decided using spacer blocks. A slight lateral laxity is accepted, but if there is excessive lateral looseness or if the spacer block cannot be inserted into to the medial gap, then a tibial recut is reconsidered. Postoperatively, immediate unlimited weight-bearing and range-of-motion exercises were started if tolerable.

Long leg radiographs were taken two weeks postoperatively, and HKA, LDFA, and MPTA were measured and patients were systematically evaluated during the immediate postoperative period, at three months, at six months, at one year, at two years, and at three years. The JLO and aHKA were calculated by MPTA + LDFA and MPTA - LDFA, respectively, and coronal plane alignment of the knee (CPAK) classification was performed using the JLO and aHKA [17]. Moreover, the restriction-based CPAK (RbCPAK) [20], where the boundaries of JLO and aHKA were decided based on the safe range of rKA-TKA, was applied to evaluate whether the restriction had worked well after the procedure. The clinical outcomes were evaluated, and the OKS, TAS, KSS-K, KSS-F, and Forgotten Joint Score 12 (FJS-12) were recorded preoperatively and three years postoperatively.

Statistical analysis

The data were collected and compiled in Microsoft Excel (Microsoft Corp., Redmond, WA). Descriptive statistics were used to present the data. Qualitative variables were expressed as frequency and percentages, while quantitative variables were expressed as mean and standard deviation. The paired t-test was used to compare the preoperative and postoperative scores. Categorized variables were compared using Fisher’s exact test. Significance level was fixed as 5% (α = 0.05). All statistical analyses were performed using easy R, an application running on R [21].

## Results

This study included 67 knees in 43 patients (Table 2).

**Table 2:** Patient demographics

Demographic details	
Patients	67 knees in 43 patients
Sex	Men 10/women 33
Age	74.1 years ± 7.7 years
Height	154.6 cm ± 7.9 cm
Weight	61.2 kg ± 10.4 kg
BMI	25.7 ± 4.4
Brand of implant	Persona CR 48 knees, GMK Sphere 19 knees

All clinical and radiographical measurements are shown in Table 3.

**Table 3:** Preoperative and postoperative values of clinical and radiographical measurements. OKS; Oxford knee score, TAS, Tegner activity score; KSS-K, Knee Society Score (knee score); KSS-F, Knee Society Score (functional score); FJS-12, Forgotten Joint Score 12; LDFA, lateral distal femoral angle; MPTA, medial proximal tibial angle; mHKA, mechanical hip-knee-ankle angle; JLO, joint line obliquity; aHKA, arithmetic hip-knee-ankle angle

	Preoperative	Postoperative	P-value (paired t-test)
Extension	-6.2° ± 7.0°	-0.1° ± 1.2°	<0.001
Flexion	129.0° ± 17.3°	128.2° ± 14.4°	0.34
OKS	27.1 ± 7.9	38.8 ± 8.3	<0.001
TAS	1.1° ± 1.0°	2.3° ± 1.2°	<0.001
KSS-K	64.7° ± 15.8°	92.2° ± 10.6°	<0.001
KSS-F	57.6 ± 25.8	74.0 ± 18.2	<0.001
FJS-12	-	61.8 ± 21.3	-
LDFA	89.0° ± 3.4°	88.6° ± 3.0°	0.20
MPTA	84.3° ± 3.1°	86.5° ± 2.7°	<0.001
mHKA	-9.2° ± 7.5°	-2.5° ± 3.8°	<0.001
JLO	173.2° ± 4.4°	175° ± 4.4°	0.001
aHKA	-4.7° ± 4.8°	-2.0° ± 3.7	0.01

All clinical scores were significantly improved three years postoperatively (p<0.001). Flexion contracture was also improved (p<0.001), but flexion angle was not improved (p=0.34).

**Figure 2:**
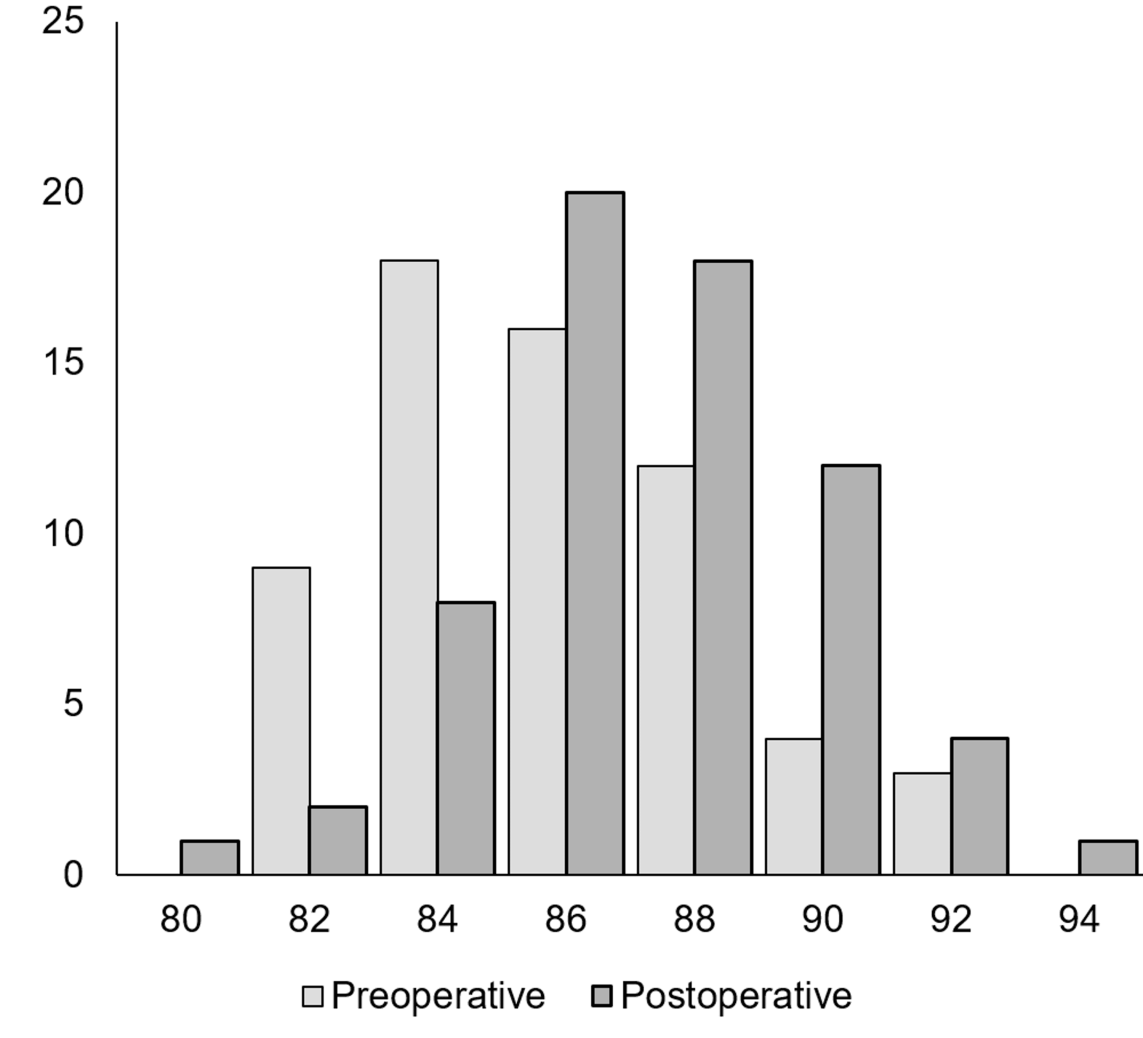
Distribution of pre- and postoperative MPTA MPTA, medial proximal tibial angle

The varus angulation was significantly corrected towards neutral in MPTA, JLO, and mHKA (p<0.001, p=0.001, p<0.001, respectively), whereas LDFA was not changed significantly (p=0.2). Distribution of MPTA is shown in Figure 2. Preoperatively, 23 (32%) knees were within the boundary, but significantly more knees (44 knees, 65%) were within the range postoperatively (p<0.001).

**Figure 3:**
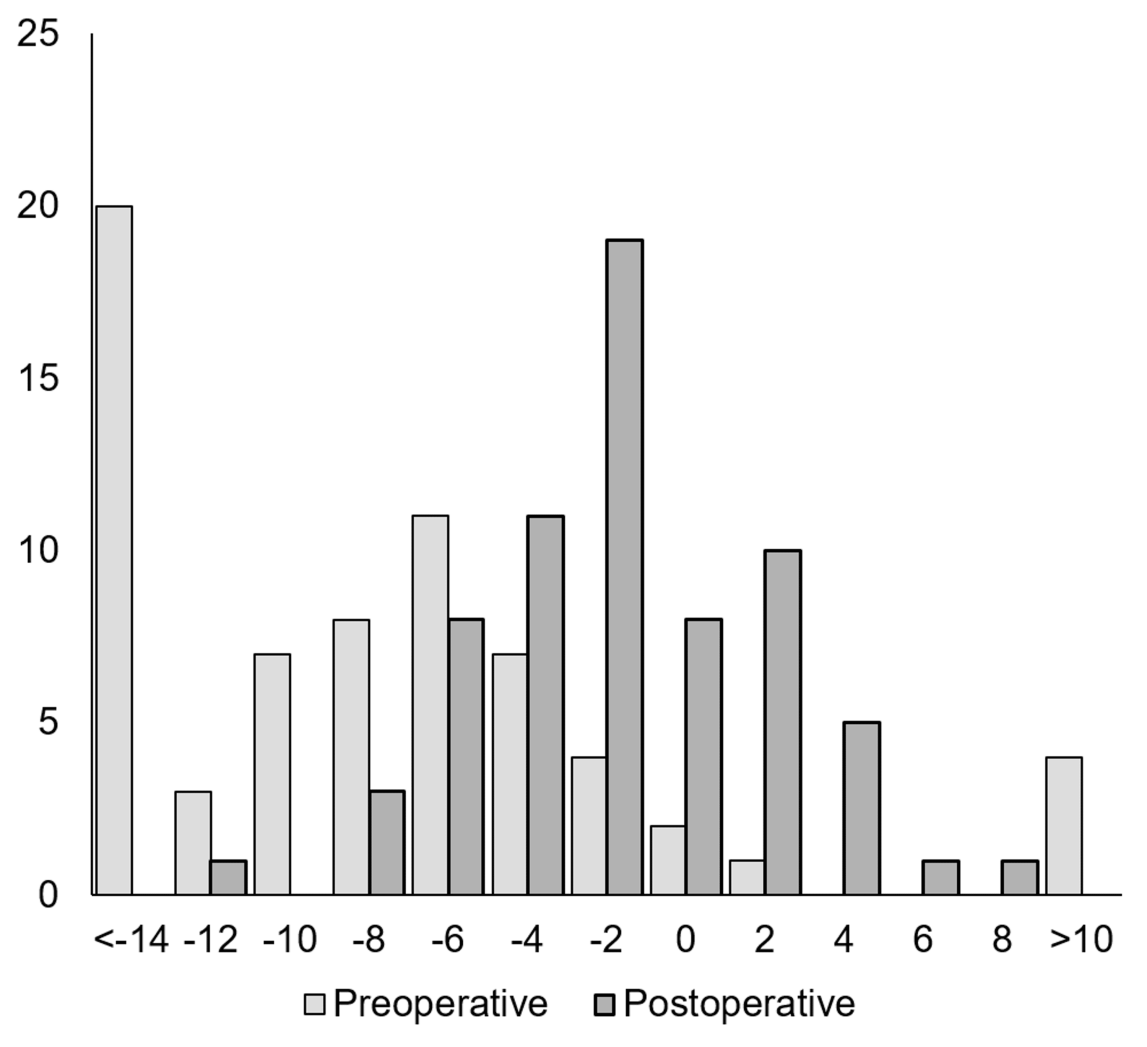
Distribution of pre- and postoperative mHKA mHKA, mechanical hip-knee-ankle axis

Distribution of mHKA was also significantly corrected within the boundary (p<0.001), from 12 knees (18%) preoperatively to 47 knees (70%) postoperatively. On the other hand, the LDFA fell within the boundary in 35 out of 67 knees. Regarding aHKA, which can express the constitutional alignment and the target of postoperative alignment after unrestricted KA-TKA, the distribution was between preoperative and postoperative mHKA, and 31 (46%) knees fell within the boundary (Figure 3).

The postoperative aHKA was also significantly smaller than the preoperative aHKA (p<0.001). Moreover, the postoperative aHKA was significantly smaller than the postoperative mHKA (p=0.01). The relationship between postoperative clinical scores and postoperative change in MPTA and mHKA is shown in Table 4. A larger mHKA and change in mHKA were associated with higher clinical scores.

**Table 4:** Pearson’s correlation coefficient change in mHKA and MPTA OKS; Oxford knee score, KSS-K, Knee Society Score (knee score); KSS-F, Knee Society Score (functional score); mHKA, mechanical hip-knee-ankle axis; MPTA, medial proximal tibial angle

	mHKA				MPTA			
	r	Upper	Lower	p-Value (t-test)	r	Upper	Lower	p-Value (t-test)
OKS	0.339	0.108	0.536	0.00499	0.213	-0.0285	0.431	0.0833
Tegner	0.304	0.0693	0.508	0.0123	0.145	-0.0988	0.372	0.242
KSS-K	0.26	0.0214	0.471	0.0334	0.198	-0.0446	0.418	0.109
KSS-F	0.136	-0.108	0.364	0.273	0.061	-0.182	0.297	0.624

Regarding CPAK classification, around 70% of knees were classified as type 1. Type 1 knees were decreased by 20%, and neutral knees in terms of both aHKA and JLO were increased postoperatively. As a result, 15 (22%) knees and 25 (37%) knees stayed in the same CPAK and restriction boundary-based CPAK, respectively (Table 5). All implants survived and no knees required revision surgery.

**Table 5:** Preoperative and postoperative distribution of CPAK and RbCPAK classification CPAK, coronal plane alignment of the knee; RbCPAK, restriction boundary-based coronal plane alignment of the knee

	Varus			Neutral			Valgus		
Apex distal	I			II			III		
		CPAK	RbCPAK		CPAK	RbCPAK		CPAK	RbCPAK
	Pre	48 (71%)	3 (4%)	Pre	11 (16%)	5 (7%)	Pre	1 (1%)	1 (1%)
	Post	21 (31%)	0 (0%)	Post	20 (30%)	5 (7%)	Post	5 (7%)	1 (1%)
Neutral	IV			V			VI		
		CPAK	RbCPAK		CPAK	RbCPAK		CPAK	RbCPAK
	Pre	3 (4%)	27 (40%)	Pre	0 (0%)	24 (36%)	Pre	0 (0%)	1 (1%)
	Post	11 (16%)	9 (13%)	Post	7 (10%)	41 (61%)	Post	7 (10%)	0 (0%)
Apex proximal	VII			VIII			IX		
		CPAK	RbCPAK		CPAK	RbCPAK		CPAK	RbCPAK
	Pre	3 (4%)	0 (0%)	Pre	1 (1%)	3 (4%)	Pre	0 (0%)	3 (4%)
	Post	1 (1%)	1 (1%)	Post	0 (0%)	6 (9%)	Post	1 (1%)	4 (6%)

## Discussion

The foremost observation of our study was that no revision was required after our CrKA-TKA. To the best of our knowledge, this is the first study of rKA (calipered technique) in a Japanese population. There are increasing numbers of mid- to long-term follow-up studies that show sufficient long-term implant survival irrespective of unrestricted or restricted TKA. Howell et al. showed implant survival at 16 years of follow-up of unrestricted KA-TKA as 93%, regardless of the postoperative whole leg and implant alignments [22]. Vendittoli et al. reported 10 years of survival rate after restricted KA-TKA [23]. There are, however, a limited number of studies on Asian populations. One study from Korea showed a 98.8% survival rate at 10 years after caliper verified unrestricted KA-TKA [24]. Nevertheless, varus phenotype of the knees is more prevalent in Asian countries than in Western countries [12,13]. rKA is therefore still considered to be safe for such populations, while for wider application, rKA without computer-assisted surgery is preferable. This is the first study to show good mid-term results after CrKA-TKA.

We report that no revisions were required and that there was a 100% survival rate up to three years after CrKA-TKA. According to the national joint registries, the revision rates at three years have been reported to be around 1-3% [1,2,25]. Although it is not possible to directly compare results, our results indicate that CrKA-TKA does not induce early implant failure, at least in the short term.

In our series, MPTA and LDFA were corrected within the boundaries in 65% and 55%, respectively. As a result, HKA was corrected within the boundaries in 70% of knees. There have been no previous reports including these or similar percentages. However, the cutting error, even after computer-assisted surgery, such as robotics, navigation, and patient-specific instrumentation, has been reported to be approximately 1°, and around 10-20% of patients fell out of ±3° even after the use of patient-specific instrumentation. Considering these results, our caliper-based technique is not thought to be perfect; however, taking into account the absence of revision cases, it is considered to be within an acceptable range.

Our series includes some extreme cases with MPTA up to 11° varus and HKA up to 13° varus. Despite the malalignment, no sign of loosening was found, and the clinical results were thought to be excellent. Although a longer follow-up is required, such alignment does not affect the postoperative outcome and implant survival, at least in the short term. Similar results were reported in previous reports of unrestricted KA-TKA [26]. Careful follow-up of such extreme alignment may discern boundaries for failure.

We report significant improvement in clinical outcomes postoperatively. Many studies have shown that KA-TKA provides similar improvement of clinical outcomes to MA-TKA [27,28], and some studies reported superior results in KA [28,29]. Although our results were those of a single-arm study, they are thought to be compatible with those of previous reports. Good clinical results can be expected with our technique without increasing the risk of component failure.

Interestingly, we found positive correction between improvement change in mHKA and postoperative TAS, OKS, and KSS-K. Moreover, there were no correlations between change in MPTA and postoperative clinical outcomes. Clinical outcome and alignment change have not been reported in previous studies. Although the correlations were not strong, the results indicate that this procedure is quite effective, even in advanced osteoarthritic knees and corrective osteotomy of tibia, which is common within the Japanese population [14], and it may not influence the clinical outcome.

Preoperatively, around 70% of knees were classified as CPAK type 1. In Western countries, it has been reported to be 20% to 30% [17], whereas more than 50% are classified as type 1 in Asian countries such as Japan [30]. Considering the racial difference, 70% of type 1 was thought to be too large compared with the previous report. However, UKA was performed in 60% of knees in all arthroplasties during this period. Most candidates for TKA were in a late stage, which might have had a bone defect or extra-articular deformity. Such cases of type 1 were dispersed to types 2, 4, and 5 postoperatively, indicating the effect of restriction. The effect of restriction is more effectively expressed using restriction-based CPAK because the boundaries of JLO and aHKA are defined based on the boundaries of the restriction protocol. As a result, around 60% of knees fell into type 5, which is considered to be within the safe zone. Theoretically, all knees are categorized into type 5 after rKA-TKA. Although there have been no previous reports regarding how many knees fell into the safe range after rKA-TKA using computer-aided devices, the results seem to be satisfactory considering that computer-assisted surgery was not used and that there was no failure.

Limitations

Limitations of this study include the short duration of follow-up, which was three years. Long-term follow-up and analysis are required to strengthen and establish the outcomes of KA-TKA. Moreover, the study is a prospective observational study and consists of patients who underwent CrKA-TKA, and there was no comparison between KA and mechanical alignment or any other alignment techniques. Also, this is a single-center study, and the surgeries were all performed by a single surgeon; hence, there is a potential for bias stemming from variations in surgeon expertise, patient selection criteria, and center-specific practices. Our sample size was 43 patients, which is considered to be small; thus, larger multicenter randomized controlled trials with this technique are required to establish the findings of this study. Finally, this was a single-arm study without a control group; hence, it is difficult to determine whether our technique yields better outcomes than standard or alternative techniques.

## Conclusions

CrKA-TKA has excellent-to-good functional outcomes and patient satisfaction within our Japanese cohort. In the three-year follow-up, there have been no revision surgeries, which dispels the concerns of implant survivorship, at least in the short term. Further long-term multicentric comparative studies examining the different alignment principles are required to draw solid conclusions.
